# Predicting Individuals' Learning Success from Patterns of Pre-Learning MRI Activity

**DOI:** 10.1371/journal.pone.0016093

**Published:** 2011-01-14

**Authors:** Loan T. K. Vo, Dirk B. Walther, Arthur F. Kramer, Kirk I. Erickson, Walter R. Boot, Michelle W. Voss, Ruchika S. Prakash, Hyunkyu Lee, Monica Fabiani, Gabriele Gratton, Daniel J. Simons, Bradley P. Sutton, Michelle Y. Wang

**Affiliations:** 1 Beckman Institute, University of Illinois at Urbana-Champaign, Urbana, Illinois, United States of America; 2 Department of Electrical and Computer Engineering, University of Illinois at Urbana-Champaign, Urbana, Illinois, United States of America; 3 Department of Psychology, The Ohio State University, Columbus, Ohio, United States of America; 4 Department of Psychology, University of Illinois at Urbana-Champaign, Urbana, Illinois, United States of America; 5 Department of Psychology, University of Pittsburgh, Pittsburgh, Pennsylvania, United States of America; 6 Department of Psychology, Florida State University, Tallahassee, Florida, United States of America; 7 Department of Bioengineering, University of Illinois at Urbana-Champaign, Urbana, Illinois, United States of America; 8 Department of Statistics, University of Illinois at Urbana-Champaign, Urbana, Illinois, United States of America; Kyushu University, Japan

## Abstract

Performance in most complex cognitive and psychomotor tasks improves with training, yet the extent of improvement varies among individuals. Is it possible to forecast the benefit that a person might reap from training? Several behavioral measures have been used to predict individual differences in task improvement, but their predictive power is limited. Here we show that individual differences in patterns of time-averaged T2*-weighted MRI images in the dorsal striatum recorded at the initial stage of training predict subsequent learning success in a complex video game with high accuracy. These predictions explained more than half of the variance in learning success among individuals, suggesting that individual differences in neuroanatomy or persistent physiology predict whether and to what extent people will benefit from training in a complex task. Surprisingly, predictions from white matter were highly accurate, while voxels in the gray matter of the dorsal striatum did not contain any information about future training success. Prediction accuracy was higher in the anterior than the posterior half of the dorsal striatum. The link between trainability and the time-averaged T2*-weighted signal in the dorsal striatum reaffirms the role of this part of the basal ganglia in learning and executive functions, such as task-switching and task coordination processes. The ability to predict who will benefit from training by using neuroimaging data collected in the early training phase may have far-reaching implications for the assessment of candidates for specific training programs as well as the study of populations that show deficiencies in learning new skills.

## Introduction

People vary in their ability to improve cognitive and psychomotor performance with practice and training. Cognitive tests predict who will benefit from training [1], [2], but they usually account for only a small proportion of the variance among individuals [3]. Here we use brain magnetic resonance imaging (MRI) data to predict individual learning success with unprecedented accuracy. Specifically, we show that patterns of time-averaged T2*-weighted images in the dorsal striatum at the start of training in a complex video-game account for more than half of the variance in the amount of subsequent learning among individuals.

In our analysis we focused on the dorsal striatum, consisting of the caudate nucleus and the putamen, and on the nucleus accumbens in the ventral striatum because of these structures' involvement in learning and execution of complex responses. The dorsal striatum plays a role in procedural and habit learning and in carrying out or initiating complex goal-directed tasks such as task-switching or reaction-time tasks [4], [5], [6], [7], [8], [9], [10]. The ventral striatum, typically related to reinforcement and motivation [8], [11], [12], is also recruited during early stages of learning [13], [14], [15]. Both the dorsal and ventral striatum show increased release and binding of dopamine, which has been associated with better performance in a video game [16]. Furthermore, an increase in the functional activity in the striatum has been associated with the transfer of updating skills in working memory tasks, possibly regulated by dopaminergic modulation [17].

With a few exceptions (e.g., the volumetric study by Erickson et al. [6]), learning has so far mostly been investigated with functional MRI (fMRI), making use of contrasts in the blood-oxygen-level dependent (BOLD) effect [18]. Measured with gradient-echo echo planar imaging (EPI), functional BOLD activity is obtained by contrasting the EPI images of an experimental condition of interest with those of a baseline condition. This emphasizes the differences between the two conditions and eliminates the part of the BOLD signal that they have in common. Here we focus on the common part, which we obtain by averaging the EPI volumes over time. The result is a time-averaged T2*-weighted image. Unlike the T1-weighted magnetization prepared rapid acquisition gradient echo (MPRAGE) image, which reflects the tissue's proton density, the T2*-weighted image depends mostly on the magnetic susceptibility of the tissue. Using multi-voxel pattern analysis (MVPA) we identified patterns of time-averaged T2*-weighted activity that predict subjects' future improvements in playing a complex video game with high accuracy.

## Results

**Figure 1:**
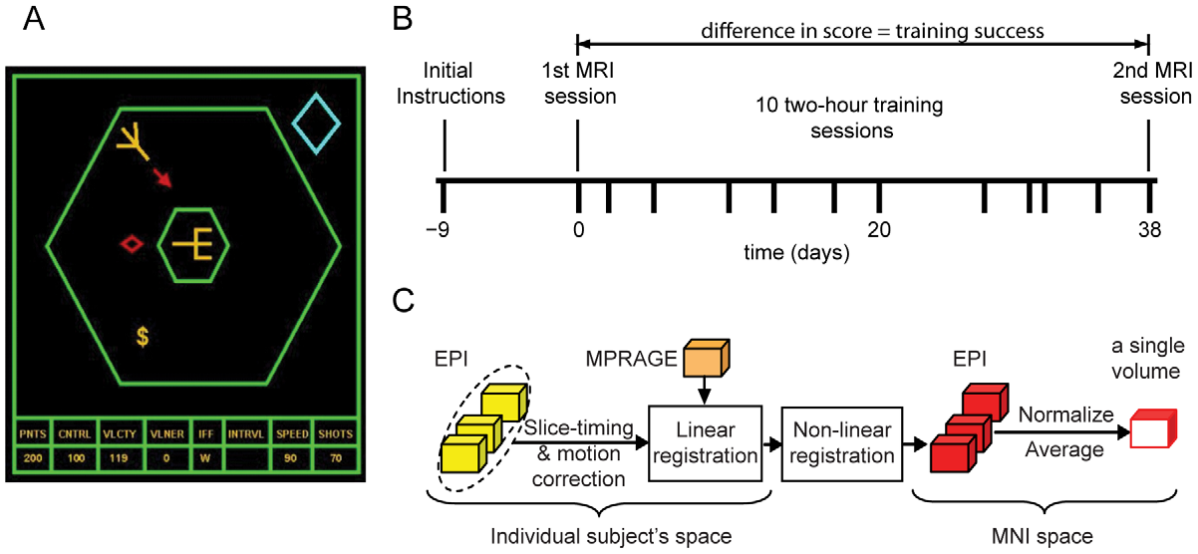
Space Fortress game, experimental time line and pre-processing flow. (A) Schematic interface of the Space Fortress video game. The objective of the game is to destroy the space fortress (yellow, center of the display) by shooting missiles at it from a space ship (yellow, upper-left corner), while moving the space ship inside the hexagon with thruster commands to evade mines (red diamond) and to collect resources (‘$’ sign). (B) Timeline of the experiment for a typical participant. After initial instructions, participants played Space Fortress in the MRI scanner while their brain activity was recorded. Next, participants underwent a total of 20 hours of training, followed by a second MRI session. We used the difference in total game score between the two MRI sessions (i.e. the score improvement) as a measure of learning success. (C) MRI preprocessing workflow: EPI volume series (1^st^ MR session) of different subjects are registered to the common space (MNI space) by linear and non-linear registration. After normalization, temporal averages of the EPI volumes are used for the subsequent analysis.

Thirty-four young adults with little experience in playing video games were trained to play Space Fortress (Figure 1A), a complex video game developed as a test bed to study skill acquisition and learning [19], [20] (see details in the Materials and Methods section). After an initial instruction session to familiarize participants with the game controls and objectives, they played Space Fortress inside an MRI scanner with an MR-compatible joystick. We recorded high-resolution anatomical T1-weighted MRI scans with an MPRAGE sequence as well as T2*-weighted images with a gradient-echo EPI sequence. The total game score during this first session inside the scanner was used as a measure of participants' abilities prior to extensive training. Over the course of the next three to eight weeks (38 days on average) participants completed ten two-hour training sessions playing Space Fortress outside the scanner (Figure 1B). Following these 20 hours of training, participants underwent a second MRI session identical to the first. The score improvement from the first to the second MRI session, i.e., the difference between the game scores in MRI sessions 2 and 1, served as a measure of individual learning success. Note that we only consider game performance during the two MRI scans in this paper, since the main focus of the paper is on predicting learning success from imaging data. For details of the progression of training outside the scanner see reference [21].

The T2*-weighted images acquired for each participant during MRI session 1 were registered linearly (7 degrees of freedom) to the T1 volume recorded in the same session. Next, a non-linear transformation was computed from the high-resolution T1 volumes to the standard Montreal Neurological Institute (MNI) space. The concatenation of these two transformations was then applied to register each subject's T2*-weighted images into MNI space. This registration was followed by a normalization step to account for variations of scanner settings between runs. The resulting T2* volumes were averaged over 16 minutes of active game play in order to suppress signal variations due to functional activity and other sources of noise. We then performed two different types of region-of-interest (ROI) based analysis with this average T2* signal to predict subjects' score improvement: spatial mean activity analysis and multi-voxel pattern analysis (MVPA). Unlike the spatial mean analysis, MVPA utilizes the distributed pattern of voxel activity within an ROI.

**Figure 2:**
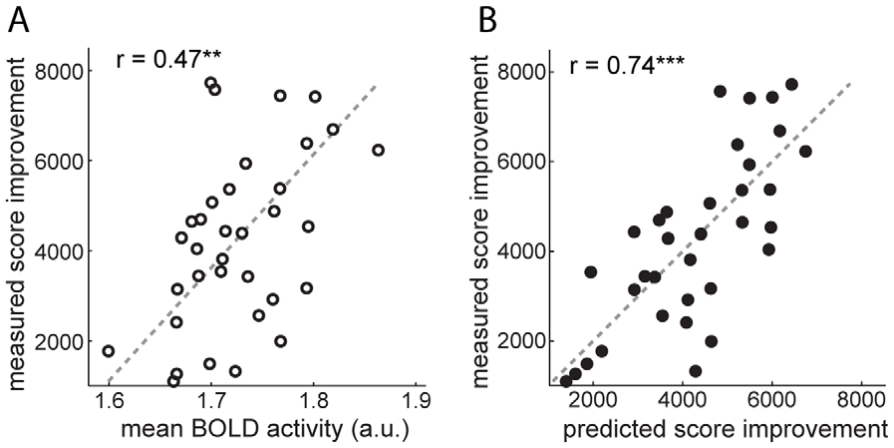
Predicting score improvement from MRI activity in the dorsal striatum. (A) Correlation of measured score improvement with the spatial mean of the time-averaged T2*-weighted signal in the dorsal striatum. Mean activity of 34 subjects is significantly correlated with score improvement. (B) Correlation of measured score improvements with score improvement predicted from multi-voxel patterns of the T2*-weighted signal in the dorsal striatum. It shows an even higher correlation than in A). The dashed lines show the least-squares best linear fits in figures A and B. **p<0.01, ***p<0.001.

For the spatial mean activity analysis, we averaged the intensity of all voxels inside an anatomically defined region. As a first test, we divided subjects into groups of good and poor learners based on a median split of their score improvements. We found significantly higher mean activity for good than poor learners in the dorsal striatum (p = 0.011), but not in the ventral striatum (p = 0.75, two-sample t tests with *n*1 = *n*2 = 17). To determine the relationship between subjects' numerical score improvements and mean activity within an ROI we computed their Pearson correlation. In the dorsal striatum, the correlation was significant (r = 0.47, p = 0.0053; see Figure 2A), but again not in the ventral striatum (r = −0.09).

**Figure 3:**
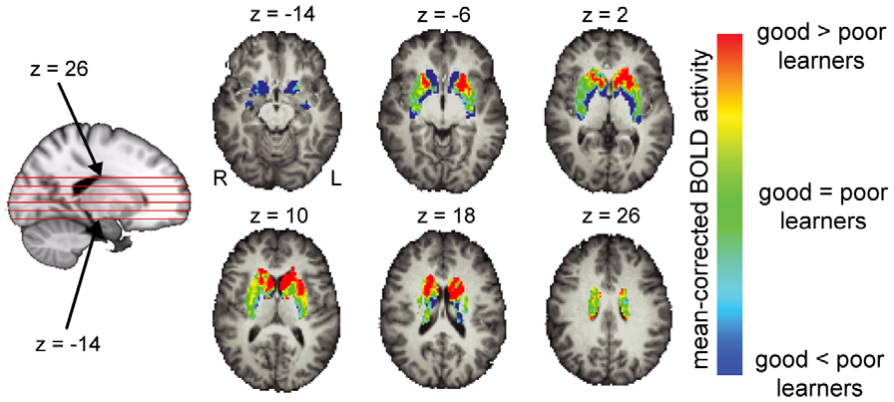
Pattern of differences between good and poor learners. Differences in activation patterns in the dorsal striatum between good and poor learners overlaid on top of six anatomical slices with z-coordinates respectively, −14, −6, 2, 10, 18, and 26. For this visualization the group of 34 subjects was split into 17 good and 17 poor learners based on the median of score improvements in Space Fortress over the course of 20 hours of training. Each subject's mean activity was subtracted from her or his activity in the dorsal striatum. The activity patterns were then averaged separately for good and poor learners. The figure shows the difference between the average patterns of good and poor learners.

Although analysis of spatial mean activity can predict score improvements to some extent, it provides merely summary statistics of the activity in an ROI, ignoring subtle differences in activity patterns. Indeed, after subtracting out each individual's average activity, good and poor learners differed in the multi-voxel patterns of time-averaged T2* activity in the dorsal striatum (Figure 3). The color patches in Figure 3 suggest a subdivision of the dorsal striatum roughly along the anterior-posterior line. In other words, good and poor learners not only differ in their level of mean activity in the dorsal striatum, but also in the local activity patterns within the dorsal striatum. These differences allow us to predict learning success for individual participants from the patterns of the temporally compounded EPI images recorded at the beginning of training with much higher accuracy than from the spatial mean of activity alone.

To exploit these differences in a multivariate analysis, we first excluded data from one subject and used activity patterns of the voxels from the remaining subjects, together with their score improvements, to train a support vector regression (SVR) algorithm [22], [23]. The algorithm then generated a prediction for the performance improvement of the excluded subject from her or his pattern of time-averaged T2*-weighted activity. The procedure was repeated so that each subject was excluded once in a leave-one-subject-out (LOSO) cross validation procedure, thereby generating predictions for each subject based on the performance and activity patterns of the other subjects. Details about the SVR algorithm and the LOSO procedure can be found in the Materials and Methods section.

The algorithmically predicted score improvements were then correlated with the actual performance improvements in Space Fortress to determine the prediction accuracy. Figure 2B shows that the predictions based on pre-training activity patterns in the dorsal striatum were highly correlated with the actual improvements that resulted from 20 hours of training (Pearson correlation coefficient *r* = 0.74, *p* = 6.1·10−7). Activity patterns before training accounted for more than half of the variance (*R*2 = 0.55) among individuals in how much they benefited from training. This represents a substantial improvement in prediction accuracy compared with the spatial mean analysis over the same regions of interest, which explained less than a quarter of the variance (22%; *r* = 0.47; Figure 2A).

**Figure 4:**
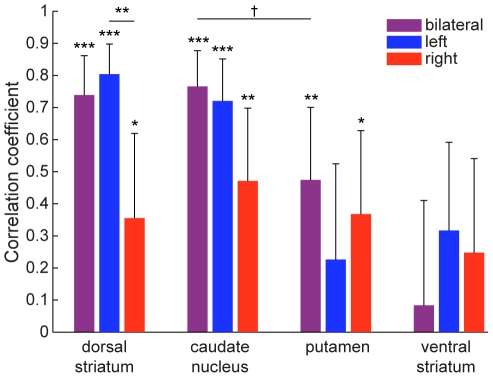
Accuracy of predicting individual score improvement from MVPA of the time-averaged T2*-weighted signal. In the dorsal striatum, correlation of predicted and measured score improvement for 34 subjects was highly significant. Within the dorsal striatum, correlation for pattern analysis was just as high in the caudate nucleus, but lower in the putamen. Predictions were even less accurate in the ventral striatum (nucleus accumbens). In the dorsal striatum, predictions were significantly more accurate based on activity patterns in the left than in the right hemisphere. The caudate nucleus showed similar lateralization, whereas the putamen did not show strong lateralization. †p = 0.051, *p<0.05, **p<0.01, ***p<0.001.

Within the dorsal striatum, predictions based on the pattern of activity in the caudate nucleus (*r* = 0.77, *p* = 1.3·10−7) were more accurate than those based on activity in the putamen (*r* = 0.47, *p* = 0.0046; Figure 4), with a marginally significant difference (*p* = 0.051). Furthermore, the left dorsal striatum (*r* = 0.80, *p* = 1.0·10−8) showed significantly higher (*p* = 0.0037) predictive power than the right dorsal striatum (*r* = 0.36, *p* = 0.039). Since all subjects were right-handed and controlled the movements of the space ship with their right hand, this may be related to motor learning in the contralateral (left) hemisphere. In contrast to good predictions from the dorsal striatum, predictions based on activity patterns in the ventral striatum (nucleus accumbens) were not correlated with measured score improvements (*r* = 0.08).

**Figure 5:**
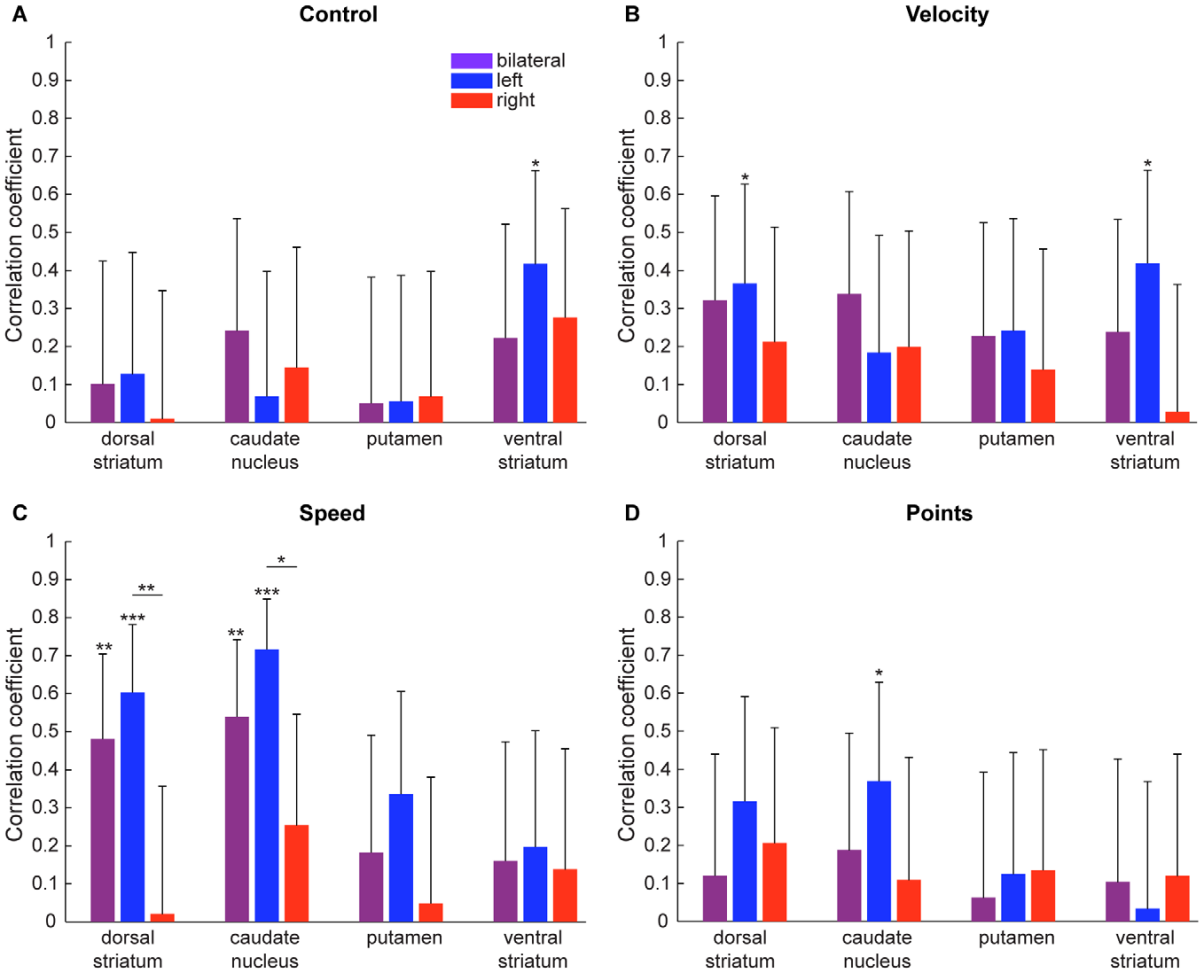
Accuracy of predicting improvements in sub-scores from the time-averaged T2*-weighted signal. (A) Improvement in the *control* sub-score is predicted to a limited extent by the time-averaged T2* activity in the left ventral striatum (nucleus accumbens). (B) The *velocity* sub-score shows small but significant correlations in the left caudate nucleus and the left nucleus accumbens. (C) Improvement in the *speed* sub-score is predicted highly significantly by time-averaged T2*-weighted activity in the dorsal striatum, in particular the caudate nucleus, but not by the ventral striatum. Correlation of predicted and measured score improvements is higher in the left than the right hemisphere. This pattern of results matches that of the total score shown in figure 4. (D) The *points* sub-score shows no significant prediction except for a small but significant correlation of predicted and measures score improvement in the left caudate nucleus. *p<0.05, **p<0.01, ***p<0.001.

The score of the Space Fortress game was composed of four sub-scores: *Control* of the space ship's position; maintaining ship *Velocity* within a predefined range; *Speed* with which subjects discriminated between and responded to different types of mines; and *Points* for successfully destroying the fortress. We repeated the SVR analysis separately for each of the sub-scores. As shown in Figure 5, the *speed* sub-score shows the same pattern of results as the total score, including the high correlation of predicted and measured score improvement in the left but not the right dorsal striatum, the higher correlation in the caudate nucleus than the putamen, and the low correlation in the ventral striatum (nucleus accumbens). This suggests that learning success with respect to discrimination and working memory (needed to identify a mine as friendly or hostile and to react to it quickly) is best predicted by time-averaged T2* activity in the dorsal striatum. Improvement in motor control, which is reflected in the *control* and *velocity* sub-scores, is not predicted to the same extent by the dorsal striatum, although both of these sub-scores are predicted at some level by T2* activity in the left nucleus accumbens. Improvements in the *points* sub-score are not predicted by activity in the striatum, except for a small but significant correlation of predicted and measured score improvement in the left caudate nucleus.

Previously, striatal brain volume was reported to predict score improvement to some extent [6], and volume of an area and its time-averaged T2* signal may be related. Another potential predictor for score improvement could be the initial score from the games played during the first MRI session. On the one hand, participants with high initial scores may already have reached ceiling performance, showing little further improvement. On the other hand, higher initial score could indicate higher cognitive abilities, enabling participants to benefit more from extensive training. To account for these factors, we used the volume of regions as reported in [6] and the initial score as two additional explanatory variables (covariates) of measured score improvements, in addition to the score improvements predicted by the SVR analysis. We used a second-order partial correlation analysis for each of the three explanatory variables to assess the unique predictive power of each of them irrespective of the other two. Table 1 shows the correlation of the SVR prediction with measured score improvement to be highly significant, even after removing the effects of striatal volume and initial score. Note that for this analysis, only those 32 of our 34 subjects were used for whom the volumetric data were available from [6]. Also, one might wonder about the use of improvement in game score during the first MRI session (e.g., from game 1 to game 4) as another predictor. However, we found no significant correlation between improvement within the first MRI session and the improvement from the first to the second MRI session (r = −0.17).

**Table 1:** Zero and second order partial correlations.

Explanatory Variables	Zero-order Pearson correlation (no covariates)	Second-order partial correlation (two covariates)
*SVR*	0.73 (p = 1.8·10−6)	0.72 (p = 3.7·10−6)
*Volumetric data*	−0.12 (p = 0.5)	0.06 (p = 0.7)
*Initial score*	−0.23 (p = 0.2)	−0.09 (p = 0.6)

Zero (Pearson correlation) and second-order partial correlations are calculated for a linear regression model with measured score improvements as the predicted variable and three explanatory variables: score improvement predicted by the SVR algorithm from time-averaged T2*-weighted activity in the dorsal striatum, volume of the dorsal striatum, and initial score.

It is important to emphasize that although we recorded the same kind of T2*-weighted EPI images that are used for functional MRI, the time-averaged EPI volumes that we used for our MVPA analysis are unlikely to be functional, because here we consider the part of the EPI images that is *common* across the time course rather than modeling the *differences* of BOLD activity over different stimulus conditions. Therefore, our signal is more likely to capture individual differences in some aspect of neuroanatomy or persistent physiology, such as differences in blood supply to the dorsal striatum or the iron concentration in this region. This view is further supported by the observation that it is not necessary to use the EPI images recorded during active game play. We obtained almost identical accuracies of predicting score improvement in Space Fortress when we use EPI images from blocks with an acoustic oddball task (r = 0.75, p = 2.9·10−7) or from blocks of passively watching Space Fortress games (r = 0.74, p = 5.6·10−7).

**Figure 6:**
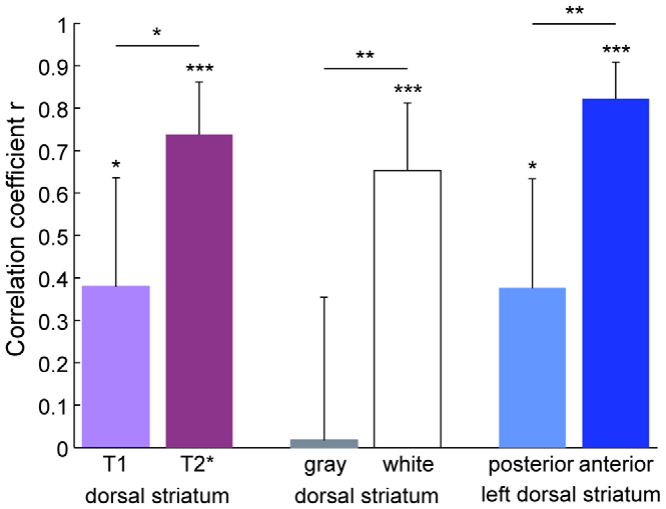
Comparison of prediction accuracy for various signal sources. Predictions based on patterns of T1-weighted images (MPRAGE) in the dorsal striatum were significantly less accurate than those based on time-averaged T2*-weighted images (EPI). Voxels located in white matter allowed for much better predictions than those in gray matter within the dorsal striatum. Finally, decoding was significantly better from the anterior than the posterior half of the left dorsal striatum. Error bars indicate the 95% confidence interval for the Pearson correlation coefficients. *p<0.05, **p<0.01, ***p<0.001.

Contrast in MR images can be obtained based on the transverse relaxation time T2 (or T2* in the case of field inhomogeneity) or the longitudinal relaxation time, T1. These two contrasts are determined by intrinsic properties of the imaged tissues. In fact, different T1 and T2 (or T2*) values help to differentiate white and gray matter in anatomical images. To test if we can predict score improvement just as well based on T1-weighted as T2*-weighted images, we subsampled the MPRAGE images that were acquired during the first scanning sessions to the same resolution as the EPI images (3.4375 mm×3.4375 mm×4 mm) and performed the MVPA analysis as described above. Correlation of predicted score improvements with measured score improvement was significantly lower for T1-weighted than T2*-weighted images (*p* = 0.031), although at 0.38 it was still significantly above zero (*p* = 0.027; Figure 6). The higher prediction accuracy in T2* compared to T1 images might hint at the importance of magnetic susceptibility of the tissue, which affects T2* but not T1. One possible source of susceptibility variations could be iron in the tissues, for instance in supplied blood [24].

Both white and gray matter contain blood vessels. In the white matter, capillaries are embedded in the myelin sheaths of axons that project over relatively long distances. In the gray matter, vessels supply mostly the somas and dendrites of neurons. Determining which tissue contributes more to the patterns that let us predict individual learning success could elucidate the anatomical and/or physiological phenomena underlying our effects. We used FSL's FAST automatic segmentation tool to separate white from gray matter in the T1 image of each individual. We then performed the LOSO cross validation analysis separately on the white matter and on the gray matter voxels (Figure 6). Correlation of predicted with observed score improvement was significantly higher (*p* = 0.0026) in the white matter (*r* = 0.65, *p* = 2.8·10−5) than in the gray matter (*r* = 0.02). This suggests that the long-range, myelinated connections in the white matter are critical for our ability to predict score improvement in Space Fortress.

In Figure 3 we had noted an apparent anterior/posterior organization of the dorsal striatum based on the difference in activity patterns between good and poor learners. To investigate this organization further, we split the left dorsal striatum in each participant with a coronal plane such that approximately equal numbers of voxels were anterior as posterior of the division. We then repeated the LOSO cross validation analysis separately for the anterior and the posterior half. Prediction accuracy was significantly higher (*p* = 0.0024) from the anterior (*r* = 0.82, *p* = 2.4·10−9) than the posterior (*r* = 0.38, *p* = 0.028) half of the left dorsal striatum (Figure 6), accounting for 68% of the variance among individuals. This result substantiates the qualitative observation in Figure 3 with a quantitative difference between anterior and posterior parts of the dorsal striatum.

## Discussion

In this study we have found that patterns of time-averaged T2*-weighted signal in the dorsal striatum recorded before the start of extensive training are highly predictive of individuals' future learning success in a complex video game (Space Fortress). Activity patterns in the dorsal striatum were by far more predictive than average activity levels (Figure 2A and 2B). Furthermore, activity patterns showed higher prediction accuracy in the left than in the right hemisphere (Figure 4), and within the left hemisphere, the anterior half of the dorsal striatum was more predictive than its posterior half (Figure 6).

The participation of the dorsal striatum in learning to play Space Fortress is consistent with its involvement in procedural and habit learning in the execution of learned behaviors (caudate nucleus) and motor learning (putamen) in non-human primates [4], [7], [8] and humans [6], [25], [26], [27]. Activity in the dorsal striatum has also been associated with tasks requiring cognitive flexibility [28] such as task-switching [9], [29] and transfer of training to untrained tasks [17]. Being associated with reward and motivation, the nucleus accumbens in the ventral striatum has also been reported to participate in early stages of learning [13], [14], [15]. However, we found patterns of time-averaged T2*-weighted signal in the nucleus accumbens not to be predictive of individual learning success.

Better performance in a video game has previously been related to an increase in dopamine release in both the dorsal and ventral striatum [16]. However, a study about the depletion of dopamine in rats [10], [30] suggested that the dopamine level in the caudate nucleus but not the nucleus accumbens was related to the initiation of complex goal-directed responses or performance, as measured by reaction time. In accordance with these reports we find that the T2*-weighted signal in a region associated with learning new skills and procedures (caudate nucleus) is more predictive of learning success than the T2*-weighted signal in sub-cortical regions associated with motor learning (putamen) or motivation and reinforcement (nucleus accumbens). As further evidence for this weighting of skills we find that improvement in the *speed* sub-score, which is related to speeded discrimination and working memory, is predicted much better by the T2*-weighted signal in the dorsal striatum than improvement in the *control* and *velocity* sub-scores, which are related to motor control.

In a previous study our group has demonstrated a link between the size of structures in the dorsal striatum and performance improvements by individual subjects [6]. Here we show that patterns of pre-learning time-averaged T2*-weighted signal can explain as much as 68% of the variance among individuals (in the anterior half of the left dorsal striatum), while volumetric analysis based on automated segmentation of these anatomical regions could explain at most 23% of the variance. However, since the volumetric measurements in [6] and the time-averaged T2*-weighted patterns used in this work both measure aspects of the same region, the dorsal striatum, they may be related. Accordingly, a partial correlation analysis of score improvement predicted by time-averaged T2*-weighted activity versus measured score improvement showed almost no additional gain by introducing two additional explanatory variables, the volume of the dorsal striatum and initial game scores (Table 1).

The ability to predict who will benefit the most from training has ramifications beyond the realm of video games. Indeed, training on Space Fortress has been associated with enhanced flight control proficiency in novice pilots [31]. In many contexts, training can be prohibitively costly and time consuming, with high attrition rates (e.g., military pilots, air traffic controllers). Pre-training MRI scans could potentially mitigate such costs by predicting who will improve at a higher rate as a result of training or to identify groups of learners who might benefit from either extended programs of training or different types of training strategies. The superior prediction power of MVPA compared to behavioral tests may justify the additional cost of MRI scans. Of course, it might also be possible, in future studies, to uncover behavioral correlates of the MRI differences, which in turn could be used to predict learning of new skills. Furthermore, our technique of applying MVPA to the temporal mean of the T2*-weighted EPI signal to predict individual differences in learning can be applied in other domains, possibly allowing for the understanding and prediction of learning as a function of development, aging, and neurodegenerative disorders. The fact that we use the gradient-echo EPI brain images, which are routinely used to measure functional activity, could make this new analysis technique especially attractive, because no new scans would need to be added to established experimental protocols. In fact, if successful in other learning contexts, the technique could be used to analyze existing data retrospectively.

Finally, the T2* signal allowed for significantly more accurate predictions than the T1 signal. This fact, along with the higher prediction rates for white than gray matter, suggests that individual differences among subjects may be due to differences in anatomical or persistent physiological features such as vascularization rather than differences in functional activation. We have recently replicated our results with an independent data set, using a similar experimental paradigm. Further experiments, including more explicit measurements of tissue susceptibility, are underway to determine the exact nature of the signal that allows for such an accurate prediction of individuals' learning success. We invite readers to comment on the possible physical and physiological interpretation of the time-averaged T2*-weighted signal that we have used in this paper.

## Materials and Methods

### Ethics Statement

The University of Illinois internal review board (IRB) approved this study, and all participants provided written informed consent according to the principles of the Declaration of Helsinki.

### Participants

Forty-two participants were recruited from the local communities of Urbana and Champaign, Illinois. All participants were young, right-handed adults between the ages of 18 and 28 with little experience with video games (less than 3 hours per week). Of the 42 participants, 39 completed the experiment, and of those 5 were excluded from the analysis because of incomplete data. The final sample consisted of 34 young adult participants (mean age  = 22, SD  = 3, 8 males) with normal or corrected-to-normal visual acuity, normal color vision, and normal hearing. At the time of data collection none of the participants were on any medications that might affect cognitive abilities. To be accepted into the study, participants were required to pass an aiming task to ensure that they were able to use the joystick.

### Space Fortress

Space Fortress (Figure 1A) was developed as a tool to study training strategies [19], [20]. Playing Space Fortress requires complex procedural learning of second-order motion control in a frictionless environment while simultaneously completing a number of other challenging tasks, including target detection and discrimination, memory updating, and resource management. Total game score is composed of four sub-scores, respectively measuring: 1. control: maneuvering the space ship in a predefined allowable area (big hexagon in Figure 1A) with thrusters, which amounts to second-order motion control in a frictionless environment without braking system; 2. velocity: keeping the velocity of the space ship within a predefined range; 3. speed: quickly and accurately handling mines, which can either be friendly or hostile; and 4. points: successfully destroying the fortress with ten missile hits with at least 250 ms separation, while preventing one's own ship from being destroyed by missiles from the space fortress or by a mine. In parallel with controlling the space ship, maintaining velocity, and handling mines and missiles, players always needed to monitor a stream of symbols for a dollar sign ($), whose second appearance indicates a bonus in the form of extra missiles or game points. In addition, players needed to retain three letters in their working memory that identified mines as friendly or hostile. The sum of these four sub-scores served as a measure of a subject's performance.

### Training procedure

Once participants had passed the aiming test, they watched an instructional video on how to play Space Fortress. After a minimal amount of practice to ensure they understood the operation of the game, participants played four 4-minute blocks of Space Fortress as part of a two-hour MRI session in a 3-Tesla Siemens Allegra MRI scanner at the Biomedical Imaging Center at the University of Illinois at Urbana-Champaign (the first MRI session). Subsequently, they played the game in two-hour training sessions (total of 20 hours), each of which consisted of 36 three-minute games. Participants underwent another MRI session identical to the first after they finished the training period (the second MRI session). The imaging data from the second MRI session were not used in any analysis described in this paper.

Score improvement was calculated as the absolute difference between the total scores of the games in the two MRI sessions. Note that it is not straight-forward to compute relative (e.g., percent) improvement, since game scores can be negative, and adding a constant offset to the score is bound to be arbitrary. We have attempted to compute relative score improvements by computing percentile ranks (R) for the game scores at time 1, and then using the mean and variance of time 1 scores to compute the percentile ranks at time 2. Relative score improvement was computed as [R(time 2) – R(time 1)]/R(time 1). Note, however, that due to the transformation to percentiles the relationship between this relative score improvement and the absolute score improvement is non-linear. Relative score improvements computed in this manner are not predicted as well by T2* activity in the dorsal striatum as absolute score improvements (r = 0.28, p = 0.11).

### MRI data acquisition

During each MRI session, an MPRAGE T1-weighted high-resolution structural volume (voxel size 1.33 mm×1.33 mm×1.30 mm, 160×192×144 voxels) was recorded for each subject. Subsequently, 13 blocks of T2*-weighted EPI images (time to echo (TE), 25 ms; repetition time (TR), 2 s; flip angle, 80°; 28 slices, 64×64 voxels matrix; voxel size, 3.4375 mm×3.4375 mm×4 mm) were acquired. The 13 blocks consisted of seven 46-second blocks of passively watching (PW) a sample video game played by an expert, interleaved with six active blocks. The six active blocks included two blocks of an odd-ball task (OB), which required counting the number of high-pitch tones among low-pitch distracters, two blocks of playing the Space Fortress game (SF block), and two blocks of playing Space Fortress while also performing the odd-ball tasks (SO block). Each active block was four minutes long. The 13 blocks were arranged in the following order: PW-OB-PW-SF-PW-SO-PW-SO-PW-SF-PW-OB-PW.

### Pre-processing

EPI data from the first MRI session were corrected for motion and slice-timing and registered linearly to the MPRAGE using FSL [32]. No explicit spatial smoothing was applied (Figure 1C). Each subject's MPRAGE image was nonlinearly registered into MNI (Montreal Neurological Institute) space using FSL's FNIRT tool. The concatenation of the linear registration from EPI to MPRAGE and the nonlinear registration from MPRAGE into MNI space was then applied to all EPI images for this subject, followed by resampling back to the resolution of the original EPI scans (3.4375 mm×3.4375 mm×4 mm). Activity in each EPI volume was divided by the mean activity in the brain of this volume to compensate for drift in scanner adjustments and differences between MRI runs and between subjects. This normalized activity was averaged over the four blocks (16 minutes) of active game play (two SF blocks and two SO blocks, 480 volumes in total). Consequently, for each subject we had one brain volume with the T2*-weighted signal aggregated over the entire period of game play inside the MRI scanner as well as the score improvement for these games.

As a control, the analysis was repeated with data from the two OB blocks as well as from the seven PW blocks. In another control, analysis was performed on the T1-weighted data. For this analysis the MPRAGE images for each subject were registered into MNI space, sub-sampled to the same resolution as the EPI images, and intensity-normalized as described above. Anatomical structures in the striatum were identified based on brain atlases included with the FSL analysis software [32].

### Support vector regression (SVR) and leave-one-out cross-validation

Support vector regression (SVR) [23] is a machine learning technique to learn the functional relationship between two types of data, *x* (in our case intensity of *d* MRI voxels) and *y* (in our case improvement in game score). Specifically, our goal here is it to use training data 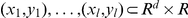 to find the coefficients *w* and offset *b* of a linear function 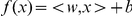 (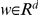 and 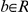) so that flatness of the coefficients *w* is maximized (i.e. minimize 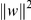), and so that no error is greater than a limit *ε*: 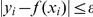. However, in order to solve the optimization problem we allow for this error condition to be relaxed by allowing some error 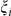 (“soft margin”), which is then penalized in the optimization. In short, we would like to solve the following optimization problem:
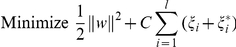


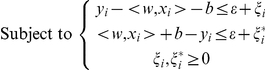
where 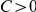
measures the trade-off between the flatness of *w* and the tolerance for deviations greater than ε.

The equivalent dual formulation of this primal objective function using Lagrange multipliers is easier to solve:
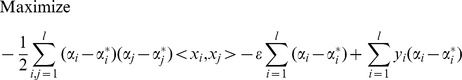



Subject to 
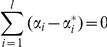
 and 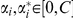, where 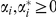 are Lagrange multipliers.

Solving the dual optimization problem, one obtains 
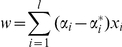
 and *b* from: 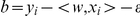 for 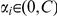 or 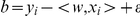 for 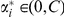
(note that 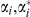 cannot be simultaneously non-zero).

As in any machine learning technique, generalization of the model parameters derived from the training data to an independent validation data set is not guaranteed. Although it is usually not possible to calculate the true error, its upper bound has been shown to be the sum of the training error and the complexity of the sets of models. For the set of hyperplanes 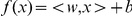, minimizing model complexity is equivalent to minimizing 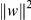 [22]. Hence, SVR allows for the derivation of the function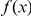, which achieves the lowest bound of the true error.

To avoid biases in the training process, the available data have to be partitioned into non-overlapping training and validation sets. This associated process of splitting the data for training and evaluating the learning is known as cross validation. Here we split the data allowing only one observation to be in the validation set. This special case of cross validation is called leave-one-out. Although this method is computationally expensive, it allows for all data to be used for training and validation in turn, while maintaining integrity of the separation of training and validation sets, thus avoiding biases in the modeling.

### Multi-voxel pattern analysis (MVPA)

We used the pre-processed imaging data and score improvements of 33 of our 34 subjects to train an SVR algorithm [22], [23] with a linear kernel. We used all voxels within an anatomical region as input features for the SVR, without any further voxel selection. Once trained with the imaging data and score improvements of the 33 training subjects, the SVR generated a prediction for the score improvement of the excluded subject based on her or his imaging data. This procedure was repeated 34 times such that each subject was excluded once. Thus, we obtained predicted score improvements for each subject in a leave-one-subject-out (LOSO) cross validation. We determined accuracy of the predictions by computing the Pearson correlation between the predicted and the measured score improvement for all 34 subjects. To test significance of correlations as well as differences between correlations we first transformed the correlation coefficients using Fisher's z transform and then performed t tests on the transformed results. The transformation is necessary, because correlation coefficients are not normally distributed, so that standard least-squares statistics cannot be applied directly. Fisher's *z* transform converts correlation coefficients to normally distributed *z* scores:
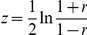



### Segmentation of white and gray matter

We used FSL's FAST tool to automatically segment the high resolution anatomical (T1-weighted) volume of each subject into white matter, gray matter, and cerebrospinal fluid (CSF). At each voxel location, FAST determined the proportion of white matter, gray matter, and CSF represented in this voxel. These partial volume maps were computed for each subject separately and then registered into MNI space at the resolution of the EPI volumes. Taking the average of these volumes over subjects gave us common segmentation maps for each of the three types of tissue. We only accepted a voxel as being white or gray matter or CSF if the respective partial volume was greater than 50% after averaging over subjects. At the end of this process we had some unassigned voxels left, for which none of the three partial volumes exceeded the 50% threshold. These voxels were excluded from further analysis. All CSF voxels lie in the cerebrospinal fluid of the lateral ventricle, whose anterior horn is immediately superior to the caudate nucleus. We did not include them in our analysis.
